# Vaccination with Recombinant *Cryptococcus* Proteins in Glucan Particles Protects Mice against Cryptococcosis in a Manner Dependent upon Mouse Strain and Cryptococcal Species

**DOI:** 10.1128/mBio.01872-17

**Published:** 2017-11-28

**Authors:** Charles A. Specht, Chrono K. Lee, Haibin Huang, Maureen M. Hester, Jianhua Liu, Bridget A. Luckie, Melanie A. Torres Santana, Zeynep Mirza, Payam Khoshkenar, Ambily Abraham, Zu T. Shen, Jennifer K. Lodge, Ali Akalin, Jane Homan, Gary R. Ostroff, Stuart M. Levitz

**Affiliations:** a Department of Medicine, University of Massachusetts Medical School, Worcester, Massachusetts, USA; b Program in Molecular Medicine, University of Massachusetts Medical School, Worcester, Massachusetts, USA; c Department of Molecular Microbiology, Washington University School of Medicine, St. Louis, Missouri, USA; d Department of Pathology, University of Massachusetts Medical School, Worcester, Massachusetts, USA; e ioGenetics LLC, Madison, Wisconsin, USA; University of Minnesota Medical School

**Keywords:** *Cryptococcus*, bioinformatics, glucans, immunization, major histocompatibility complex

## Abstract

Development of a vaccine to protect against cryptococcosis is a priority given the enormous global burden of disease in at-risk individuals. Using glucan particles (GPs) as a delivery system, we previously demonstrated that mice vaccinated with crude *Cryptococcus*-derived alkaline extracts were protected against lethal challenge with *Cryptococcus neoformans* and *Cryptococcus gattii*. The goal of the present study was to identify protective protein antigens that could be used in a subunit vaccine. Using biased and unbiased approaches, six candidate antigens (Cda1, Cda2, Cda3, Fpd1, MP88, and Sod1) were selected, recombinantly expressed in *Escherichia coli*, purified, and loaded into GPs. Three mouse strains (C57BL/6, BALB/c, and DR4) were then vaccinated with the antigen-laden GPs, following which they received a pulmonary challenge with virulent *C. neoformans* and *C. gattii* strains. Four candidate vaccines (GP-Cda1, GP-Cda2, GP-Cda3, and GP-Sod1) afforded a significant survival advantage in at least one mouse model; some vaccine combinations provided added protection over that seen with either antigen alone. Vaccine-mediated protection against *C. neoformans* did not necessarily predict protection against *C. gattii*. Vaccinated mice developed pulmonary inflammatory responses that effectively contained the infection; many surviving mice developed sterilizing immunity. Predicted T helper cell epitopes differed between mouse strains and in the degree to which they matched epitopes predicted in humans. Thus, we have discovered cryptococcal proteins that make promising candidate vaccine antigens. Protection varied depending on the mouse strain and cryptococcal species, suggesting that a successful human subunit vaccine will need to contain multiple antigens, including ones that are species specific.

## IMPORTANCE

The encapsulated fungi *Cryptococcus neoformans* and *Cryptococcus gattii* are responsible for nearly 200,000 deaths annually, mostly in immunocompromised individuals. An effective vaccine could substantially reduce the burden of cryptococcosis. However, a major gap in cryptococcal vaccine development has been the discovery of protective antigens to use in vaccines. Here, six cryptococcal proteins with potential as vaccine antigens were expressed recombinantly and purified. Mice were then vaccinated with glucan particle preparations containing each antigen. Of the six candidate vaccines, four protected mice from a lethal cryptococcal challenge. However, the degree of protection varied as a function of mouse strain and cryptococcal species. These preclinical studies identify cryptococcal proteins that could serve as candidate vaccine antigens and provide a proof of principle regarding the feasibility of protein antigen-based vaccines to protect against cryptococcosis.

## INTRODUCTION

The encapsulated yeasts *Cryptococcus neoformans* and *Cryptococcus gattii* are major causes of morbidity and mortality in persons with qualitative and quantitative impairment of CD4^+^ T cells (1, 2). Worldwide, it has been estimated that close to 200,000 people with AIDS die from cryptococcal meningitis annually (3). The lifetime incidence of cryptococcosis in recipients of solid organ transplants is approximately 3% (4). Other high-risk groups have been identified, including persons with lymphoma, sarcoidosis, and idiopathic CD4 lymphocytopenia. *C. gattii* is more of a primary pathogen than *C. neoformans*, with many cases occurring in individuals with relatively intact immune function (1, 2). Of particular concern is the emergence and spread of hypervirulent *C. gattii* strains in parts of Canada and the United States (2, 5, 6).

Exposure to *Cryptococcus* is thought to most commonly occur following inhalation of fungi into the lungs (1, 7). If innate defenses fail, then an adaptive antigen-specific, T cell-mediated immune response becomes critical for preventing local spread and dissemination. Antibody responses, particularly those directed against glucuronoxylomannan (GXM) and other polysaccharide components in the capsule, appear to contribute to the adaptive response in at least some individuals (8).

The high global burden of cryptococcosis (3) has prompted research into the development of vaccines to protect individuals at high risk. Protection of mice using live and killed vaccines has been demonstrated using genetically altered whole yeast cells (9–12). Vaccines comprised of GXM and other cell wall carbohydrates conjugated to carrier proteins elicit antibody responses to GXM that protect in experimental models of cryptococcosis (13, 14). We and others have focused on the discovery of *C. neoformans* antigens that stimulate T cell responses and thus could serve as candidate antigens in cryptococcal vaccines (15–18). Moreover, because most vaccine adjuvants presently in clinical use stimulate predominantly protective antibody responses (19), we postulate that a successful vaccine will need to incorporate adjuvants and/or delivery systems that elicit strong T cell responses.

Our laboratories have been exploring glucan particles (GPs) as a combined delivery system and adjuvant for cryptococcal vaccines. GPs are hollow, porous yeast cell wall shells produced by treating *Saccharomyces cerevisiae* with a series of alkaline, acid, and solvent extractions (20–23). They are principally composed of β-1,3-glucan polymers with about 2% chitin and undetectable amounts of proteins, lipids, and mannans. Immune recognition of GPs is mediated by Dectin-1 and complement receptors (24). Immunization of mice with GPs containing trapped ovalbumin results in robust and durable antigen-specific antibody and Th1/Th17-biased CD4^+^ T cell responses (20, 21).

Recently, we performed whole-cell mild alkali extraction of acapsular mutant strains of *C. neoformans* and *C. gattii*. The soluble extracts were loaded into GPs and used to vaccinate mice. Mice so vaccinated exhibited CD4^+^ T cell recall responses in the lungs and were partially protected from pulmonary challenge with *C. neoformans* and *C. gattii* (15). In the present study, we sought to identify specific cryptococcal proteins responsible for protection. Candidate antigens were chosen using biased and unbiased approaches, recombinantly expressed in *Escherichia coli*, and loaded into GPs. Mouse strains were vaccinated with the antigen-laden GPs and then challenged with *C. neoformans* and *C. gattii*. We found that protection varied depending on the antigen, mouse strain, and cryptococcal species.

## RESULTS

**FIG 1:**
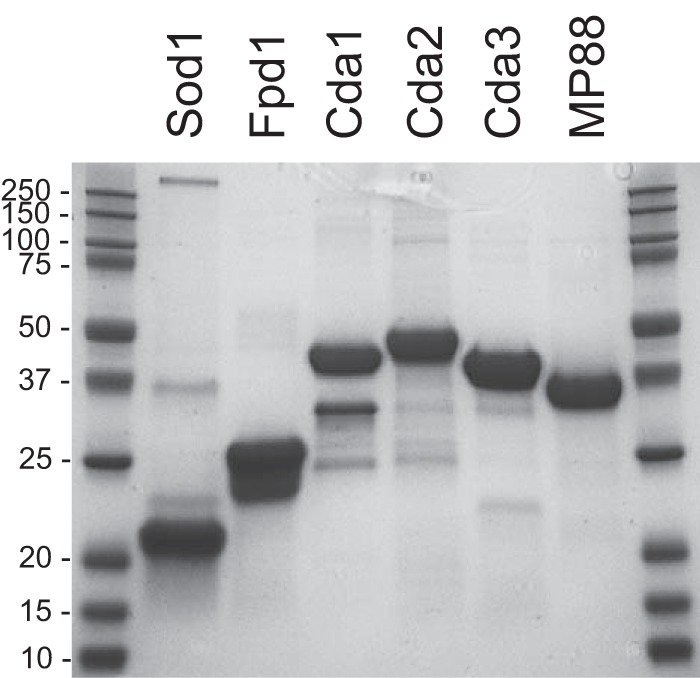
SDS-PAGE of purified recombinant proteins used to make GP vaccines. Each lane contains 10 μg of total protein. Proteins were stained with Coomassie. Molecular masses (in kilodaltons) of protein standards are indicated. The expected molecular masses of the proteins are listed in Table 1.

The six cryptococcal proteins selected for testing are described in Table 1. The immunogenic properties of three of the proteins (Cda2, Fpd1, and MP88) have previously been reported (16–18). Cda1 and Cda3 were chosen as they are chitin deacetylases (Cda) with protein sequence homology to Cda2 (25) and for the importance of Cda proteins for virulence (26). Sod1 was the most abundant protein in a mild alkaline extract that was immunoprotective (15) and contributes to virulence of *C. gattii* (27). With the exception of Sod1, the six proteins have little to no homology to human proteins. RNA encoding each of the proteins has been identified by transcriptome sequencing (RNA-seq) in *C. neoformans* obtained from human patients with cryptococcal meningitis (28). In addition, all six proteins have been identified as part of the secretome of the fungus (29, 30). The *C. neoformans* strain H99 proteins were expressed with a His tag in *E. coli* (Table 1) and purified over a nickel column. Five of the recombinant proteins were made from cDNA generated from isolated mRNA, while the cDNA for Cda3 expression was synthesized. Sequences for a predicted signal peptide (found in the full-length proteins, except for Sod1), as well as C-terminal serine/threonine-rich regions and a putative glycosylphosphatidylinositol (GPI) anchor motif (found in the proteins except Fpd1 and Sod1) were not included. Milligram quantities of each protein were purified, confirmed to be of the correct size by SDS-PAGE (Fig. 1), and loaded into GPs, typically at 10 μg per vaccine dose.

**TABLE 1:** Recombinant proteins of *C. neoformans* strain H99 expressed in *E. coli* and used for testing as vaccines

CNAG no.	Name	Description	Region expressed	Vector	Expressed protein mass (kDa)	% of identity to *C. gattii* R265 homolog^a^	Human homology^b^	No. of RNA-seq reads^c^
CNAG_05799	Cda1	Chitin deacetylase	20–374	pET200	42.3	90	None	139,907
CNAG_01230	Cda2	Chitin deacetylase	20–378	pET200	43.8	88	None	5,835
CNAG_01239	Cda3	Chitin deacetylase	19–336	pET19b	37.9	87	5.3	864
CNAG_06291	Fpd1	Polysaccharide deacetylase	20–249	pET200	29.3	92	None	1,511
CNAG_01019	Sod1	Superoxide dismutase	2–154	pET200	20.1	70	5^−62^	70,771
CNAG_00776	MP88	Mannoprotein	23–294	pET200	32.9	94	None	24,244

a *C. gattii* strain R265 protein sequences were retrieved from the Ensembl Fungi website (http://fungi.ensembl.org/index.html). Regions of *C. gattii* protein comparable to *C. neoformans* protein sequence expressed in *E. coli* were aligned to calculate the percentage of identity.

b Homology to the human protein with the highest similarity (BLASTp analysis); the smaller the numerical value, the greater the similarity.

c Shown are the average numbers of normalized RNA-seq reads recorded for two strains of *Cryptococcus* isolated directly from infected human patients (GEO GenBank accession no. GSE51573) (28).

**FIG 2:**
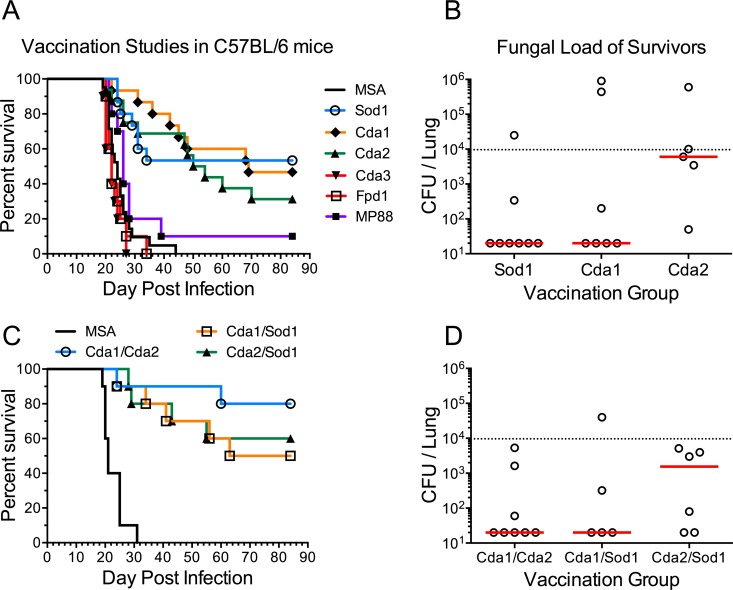
Vaccination studies with GP-recombinant protein vaccines in C57BL/6 mice. Mice were vaccinated 3 times with 10 µg/vaccine dose of the indicated recombinant protein in GPs and then challenged with 10^4^ CFU of strain KN99. Survival curves and fungal burden in the lungs of mice that survived 84 days p.i. are shown for mice vaccinated with one recombinant protein (A and B) and with two recombinant proteins (C and D). Data were combined from two to three independent vaccination studies with five to six mice per vaccination group in each study. (A) *P* < 0.001 comparing MSA with Sod1, Cda1, or Cda2. (C) *P* < 0.001 comparing MSA with any other group. In panels B and D, the dotted line indicates the CFU of KN99 challenge; median CFU per lung for each group are indicated by a solid line.

**FIG 3:**
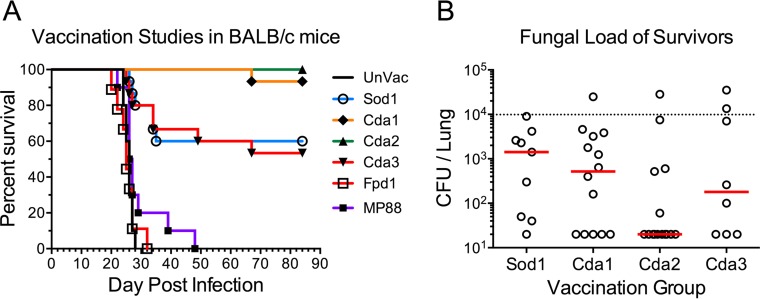
Vaccination studies with GP-recombinant protein vaccines in BALB/c mice challenged with KN99. Studies were conducted as in Fig. 2A and B, except BALB/c mice were used, and the comparator was unvaccinated (UnVac) mice. Survival curves and fungal burden in the lungs of mice that survived 84 days p.i. are shown. Data were combined from two to three independent vaccination studies with four to five mice per vaccination group in each study. *P* < 0.001 comparing UnVac with Sod1, Cda1, Cda2, or Cda3. For panel B, the dotted line identifies CFU of KN99 challenge; median CFU per lung for each group are indicated by a solid line.

The vaccines were first tested in C57BL/6 mice. Each mouse received a prime and two boosts of GPs formulated with either one of the six recombinant proteins or with mouse serum albumin (MSA) as a control. Two weeks following the last boost, the mice were challenged with *C. neoformans* strain KN99 and monitored for 84 days. Significantly prolonged survival was observed following vaccination with Sod1, Cda1, and Cda2 (Fig. 2A). Some of the survivors had undetectable lung CFU when the study was terminated (Fig. 2B). Binary combinations of Sod1, Cda1, and Cda2 in GPs were then tested, with Cda1/Cda2 showing the most promising results (Fig. 2C and D). When the vaccine candidates were tested in BALB/c mice, complete and nearly complete protection was observed with Cda2 and Cda1, respectively (Fig. 3). Partial protection was again seen with Sod1. Cda3, which did not protect C57BL/6 mice, partially protected the BALB/c mice.

**FIG 4:**
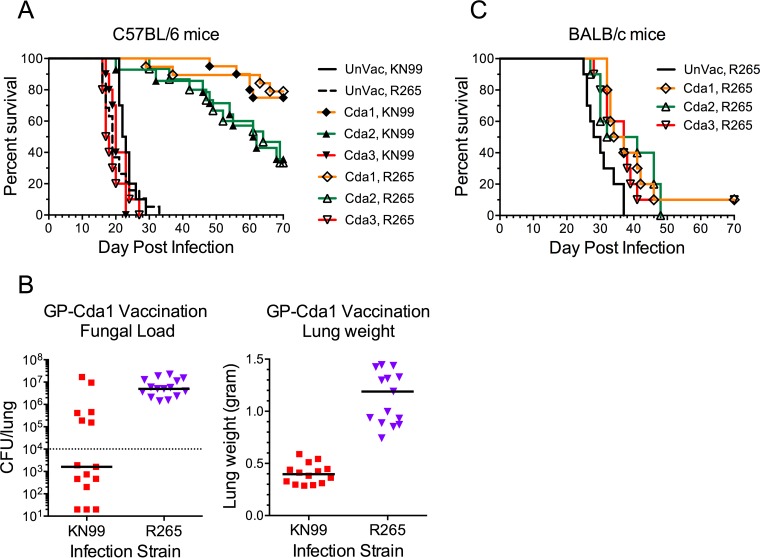
Survival of vaccinated C57BL/6 and BALB/c mice following infection with *C. gattii* strain R265. C57BL/6 (A) and BALB/c (C) mice were vaccinated 3 times with 10 µg/vaccine dose of recombinant Cda1, Cda2, or Cda3 in GPs. Control mice were left unvaccinated (UnVac). Mice were then challenged with 10^4^ CFU of KN99 (*C. neoformans*) or R265 (*C. gattii*). Panel B shows fungal burden in the lungs (left panel) and lung weights (right panel) of C57BL/6 mice vaccinated with GP-Cda1 that survived 84 days. Data were combined from two to four independent experiments, each with four to five mice per group. (A) *P* < 0.001 comparing UnVac with Cda1 or Cda2 for both KN99 and R265. (B) *P* < 0.01 and *P* < 0.001 comparing fungal load and lung weight, respectively, in KN99 with R265. (C) *P* < 0.05 comparing UnVac with any other group. In panel B, the dotted line indicates CFU of KN99 challenge; the median CFU per lung for each group are indicated by a solid line.

Next, we assessed the capacity of Cda1, Cda2, and Cda3 GP-based vaccines to protect following challenge with *C. gattii* strain R265. For C57BL/6 mice, the survival curves were nearly identical comparing lethal infections with *C. neoformans* to that with *C. gattii*; Cda1 and Cda2 were protective against both strains, whereas Cda3 was not (Fig. 4A). However, at the termination of the study, lung fungal loads and weights were significantly lower in the Cda1-vaccinated mice challenged with KN99 compared with R265 (Fig. 4B). In contrast to C57BL/6 mice, BALB/c mice challenged with R265 had only a minor prolongation of survival following vaccination with GPs containing Cda1, Cda2, or Cda3 (Fig. 4C). Moreover, BALB/c mice vaccinated with both GP-Cda1 and GP-Cda2 (Cda1/Cda2) and challenged with R265 all succumbed by day 52 postinfection (p.i.) (see Fig. S1 in the supplemental material).

10.1128/mBio.01872-17.2 FIG S1  Survival of C57BL/6 and BALB/c mice vaccinated with Cda1 and Cda2 and challenged with *C. neoformans* strain KN99 or *C. gattii* strain R265. Mice were vaccinated with a mixture of Cda1 and Cda2 as in Fig. 2C, except the vaccination dose for each antigen was 6 µg. The challenge dose of KN99 and R265 was 10^4^ CFU. (A) Survival curves of vaccinated and challenged mice. Data are from two independent experiments with 4 or 5 mice per group. *P* < 0.001 comparing C57BL/6 KN99 with C57BL/6 R265. *P* < 0.001 comparing BALB/c KN99 with BALB/c R265. Unvaccinated controls all died between 16 and 33 days postinfection (data not shown). (B) Lung fungal burdens of mice that survived for 12 weeks. For panel B, the dotted line identifies CFU of KN99 and R265 challenge; the median CFU per lung for each group are indicated by a solid line. Download FIG S1, PDF file, 0.4 MB. Copyright © 2017 Specht et al. 2017 Specht et al. This content is distributed under the terms of the Creative Commons Attribution 4.0 International license.

**FIG 5:**
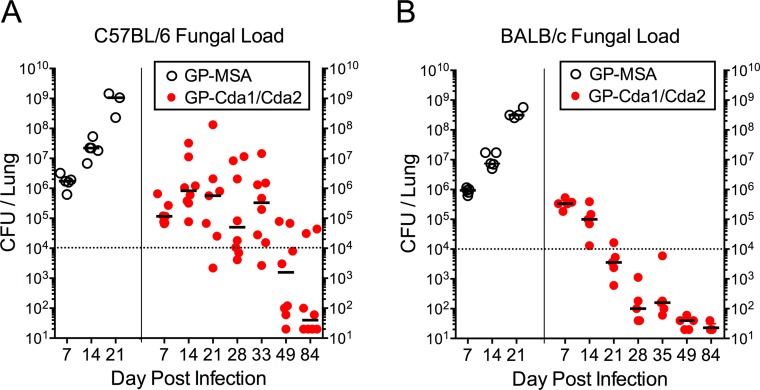
Lung CFU of C57BL/6 and BALB/c mice vaccinated with GP-Cda1/Cda2 and challenged with *C. neoformans* strain KN99. C57BL/6 (A) and BALB/c (B) mice were vaccinated three times with 10 μg/vaccine dose of GP-Cda1 and GP-Cda2 (GP-Cda1/Cda2) and then challenged with 10^4^ CFU of *C. neoformans* strain KN99. Control mice received GP-MSA. At the indicated time points, mice were euthanized and lung CFU determined. For panel A, GP-Cda1/Cda2 data were collected from three independent studies (does not include day 84 data from Fig. 2D), and for panel B, data were from two independent studies. Each datum point represents the CFU from one mouse.

In the next set of experiments, we examined pulmonary fungal loads following challenge of GP-Cda1/Cda2-vaccinated C57BL/6 and BALB/c mice with 10^4^ CFU of *C. neoformans* KN99. Control mice were immunized with GP-MSA. Mice were euthanized at selected time points. For both strains of mice immunized with GP-MSA, lung CFU rose inexorably (Fig. 5). Levels close to 10^9^ CFU/lung were seen 21 days after challenge, a time point shortly before the mice typically succumb to infection. In contrast, for mice vaccinated with GP-Cda1/Cda2, lung CFU initially rose, but then eventually declined in the day 84 survivors. However, the mouse strains differed at the intermediate time points studied, with the C57BL/6 mice showing highly variable numbers of CFU, whereas the BALB/c mice had a steady, consistent decline 7 days after challenge.

**FIG 6:**
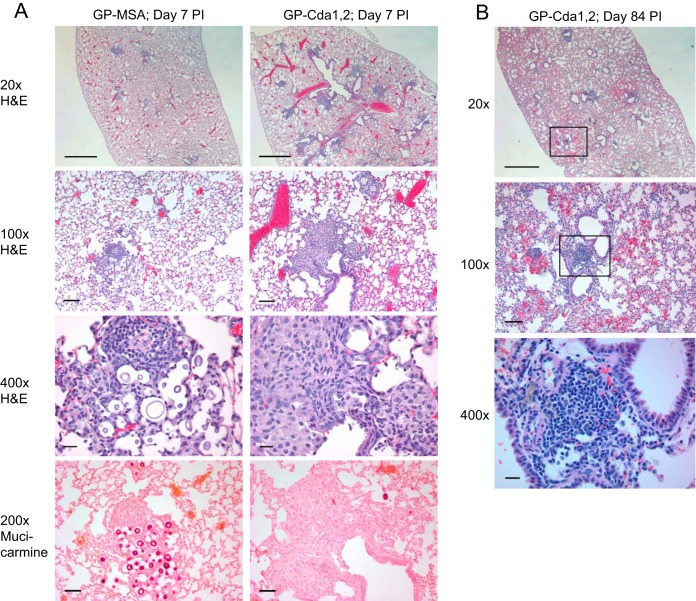
Histology of mouse lungs. Shown are representative H&E- and mucicarmine-stained sections of lungs from vaccinated mice. (A) Mice were vaccinated three times with 10 μg/vaccine dose of GP-Cda1 and GP-Cda2 (GP-Cda1/Cda2) and then challenged with 10^4^ CFU of *C. neoformans* strain KN99. Control mice received GP-MSA. After 7 (A) and 84 (B) days, mice were euthanized and lung sections prepared. Scale bars on the photomicrographs are as follows: 20× = 1 mm, 100× = 100 µm, 200× = 50 µm, and 400× = 20 µm.

Lung sections were prepared from BALB/c mice vaccinated with GP-Cda1/Cda2 or GP-MSA 7 and 14 days after challenge with KN99 and evaluated for fungal burden and extent of inflammation by a pathologist blind to experimental condition (Fig. 6A; see Fig. S2 in the supplemental material). Hematoxylin and eosin (H&E)-stained sections revealed mixed inflammatory infiltrates predominantly composed of histiocytes and small numbers of neutrophils and eosinophils within the airways, adjacent alveolar spaces, and walls in all the groups (Fig. 6A). Aggregates of lymphocytes consistent with the formation of inducible bronchus-associated lymphoid tissue (iBALT) were also noted in some of the airway walls in all groups, including the GP-MSA controls. The inflammation progressed to involve more distant airspaces and walls (in addition to airways and adjacent alveolar airspaces and walls) in mice sacrificed at day 14 compared to those sacrificed at day 7. In addition, we noted a slight increase in the proportion of neutrophils among the inflammatory infiltrates at day 14. When comparing the lung pathology at day 7 of mice vaccinated with GP-Cda1/Cda2 to that of controls that received GP-MSA, there was a trend toward greater inflammation in the vaccinated group, suggesting the vaccine promoted an earlier and more intense immune response to fungal microorganisms. No significant, morphological differences were noted in the extent of the inflammation in different mouse groups sacrificed at day 14 (Fig. S2). Mucicarmine-stained sections highlighted that yeast cells present within the airways and airspaces were surrounded by inflammatory cells (Fig. 6). Consistent with the quantitative CFU data, we noted significantly fewer microorganisms in mice vaccinated with GP-Cda1/Cda2 compared with controls that received GP-MSA on both days 7 and 14.

10.1128/mBio.01872-17.3 FIG S2  Pulmonary inflammation and fungal burden seen on pathology. BALB/c mice were vaccinated three times with 10 µg/vaccine dose each of GP-Cda1 and GP-Cda2 (Cda1/2) and then challenged with 10^4^ CFU of *C. neoformans* strain KN99. Control mice received GP-MSA (MSA). At 7 and 14 days postchallenge, mice were euthanized, and stained slides prepared from sectioned lungs were scored by a pathologist blind as to group. *n =* 5 mice/group. For fungal burdens, *P* < 0.05 and *P* < 0.001, comparing MSA with Cda1/2 at days 7 (D7) and 14 (D14), respectively. Download FIG S2, PDF file, 0.1 MB. Copyright © 2017 Specht et al. 2017 Specht et al. This content is distributed under the terms of the Creative Commons Attribution 4.0 International license.

Lung sections from mice vaccinated with GP-Cda1/Cda2 and euthanized at day 84 post-cryptococcal challenge revealed scattered aggregates of lymphocytes in the wall of the airways (iBALT) (Fig. 6B). Rare collections of alveolar macrophages with foamy cytoplasm were also noted in lung sections. However, no fungal microorganisms were identified on H&E- and mucicarmine-stained sections. There were no surviving control GP-MSA mice at day 84 to study comparatively.

**FIG 7:**
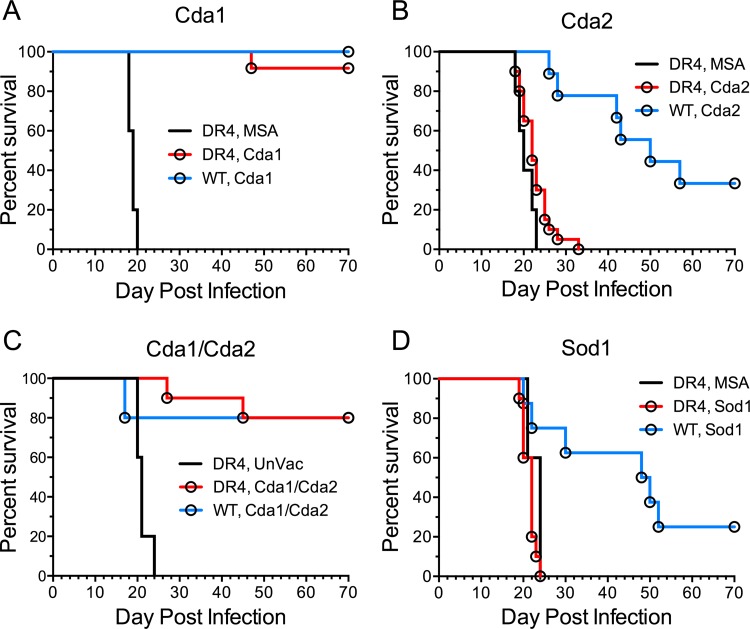
Protection of vaccinated DR4 mice from infection with *C. neoformans* strain KN99. DR4 and wild-type (WT) C57BL/6 mice were vaccinated three times with 10 μg/vaccine dose of GPs containing (A) Cda1, (B) Cda2, (C) Cda1 and Cda2 (Cda1/Cda2), and (D) Sod1. Control mice received GPs containing MSA or were left unvaccinated (UnVac). Mice were then challenged with 10^4^ CFU of *C. neoformans* strain KN99 and monitored for survival. Data from vaccinated DR4 mice were combined from at least two independent experiments with a total of 10 to 20 mice per vaccination group. The number of control mice per experimental group ranged from 5 to 10. *P* < 0.001 comparing DR4 MSA with DR4 Cda1, *P* < 0.05 comparing DR4 MSA with DR4 Cda2, and *P* < 0.001 comparing DR4 UnVac with DR4 Cda1/2.

We next assessed whether the lead vaccine candidates could protect translationally relevant humanized DR4 mice against challenge with *C. neoformans* strain KN99. DR4 mice have a C57BL/6 background, but express human major histocompatibility complex class II (MHC-II) HLA-DR4 (DRB1*0401) while lacking endogenous murine MHC-II (31). GPs containing Cda1 or the combination of Cda1 and Cda2 induced protective responses in DR4 mice that were comparable to that seen following vaccination of C57BL/6 mice (Fig. 7). However, vaccines containing Cda2 and Sod1, which significantly protected C57BL/6 mice, afforded the DR4 mice no protection.

Finally, we performed individual epitope mapping of the six proteins of interest to try to understand the mechanisms responsible for the observed differences in vaccine responses among mouse strains and to assess the potential of the proteins as candidate antigens in human vaccines (see Fig. S3 in the supplemental material). Each protein contains several high-probability B cell linear epitopes and from 1 to 3 sequences comprising strong MHC-II binding for multiple human and murine alleles. In Fig. S3A to F, high-probability B cell epitopes and predicted MHC-II high-binding peptides are indicated with a score exceeding 1 standard deviation from the mean for that protein. Within these regions, multiple peptides are also predicted to be excised by cathepsin B, L, or S (data not shown) and so have the potential for presentation as T cell epitopes by antigen-presenting cells. When examined from the perspective of individual murine MHC-II allele binding, there are differences in the predicted binding between alleles, leading to differences in epitope dominance between mice. For instance, in Cda1, Fig. S3A shows similarity between binding by H2-I-Ab (C57BL/6) and H2-I-Ad (BALB/c) for the 15-mer with its index position at position 309, but similarity between H2-I-Ad and the human DRB1:0401 is carried by the DR4 mouse at positions 291 to 292. This may result in the observed differences in survival between C57BL/6, BALB/c, and DR4 mice. Thus, all of the recombinant proteins are characterized as good potential immunogens that are likely to elicit antibody responses and sustained cellular recall responses. The engineering in of the N-terminal His tag did not apparently bias the potential immunogenicity or dominance of the epitopes of the *Cryptococcus* protein.

10.1128/mBio.01872-17.4 FIG S3  Comparison of B cell linear epitopes and human and murine MHC-II binding patterns. For each protein tested as a candidate vaccine antigen, the probability of participation in a B cell linear epitope and predicted MHC-II binding affinities of sequential 15-mer peptides are shown in the five panels. Panels should be read from top to bottom. Panel 1 shows the probability of linear B cell epitopes for both mice and humans. Panel 2 shows the permuted human MHC-II binding, smoothed across 16 DRB alleles. Panel 3 shows MHC-II binding for allele H-2-IAb (C57BL/6 mice). Panel 4 shows MHC-II binding for allele H-2-IAd (BALB/c mice). Panel 5 shows MHC-II binding for human allele DRB1-0401 found in transgenic mouse strain DR4. *y*-axis units are the inverted standard deviation of linear B cell epitope probability, and for MHC binding panels, they are standard deviation units below the mean of the natural log binding affinity (ln of the 50% inhibitory concentration [lnIC_50_]) for that protein. The vertical black line in each set of panels indicates the boundary between vector sequence that includes the His tag and each *Cryptococcus* protein sequence. The epitope predictions illustrated in panels A to F were generated using the ioGenetics’ EigenBio platform. The EigenBio algorithms are written on JMP (SAS Institute). The rationale underlying the predictions is further described in several publications by R. D. Bremel and E. J. Homan as cited in Text S1 in the supplemental material (Immunome Res 6:8, 2010, https://doi.org/10.1186/1745-7580-6-8; Immunome Res 6:7, 2010, https://doi.org/10.1186/1745-7580-6-7; PLoS One, 8:e70115, 2013, https://doi.org/10.1371/journal.pone.0070115; Front Immunol 5:541, 2014, https://doi.org/10.3389/fimmu.2014.00541). Download FIG S3, PDF file, 0.4 MB. Copyright © 2017 Specht et al. 2017 Specht et al. This content is distributed under the terms of the Creative Commons Attribution 4.0 International license.

10.1128/mBio.01872-17.1 TEXT S1  Methods specific to Fig. S3. Download TEXT S1, DOCX file, 0.1 MB. Copyright © 2017 Specht et al. 2017 Specht et al. This content is distributed under the terms of the Creative Commons Attribution 4.0 International license.

## DISCUSSION

Compared with vaccines featuring whole organisms or crude antigen preparations, subunit vaccines have the advantage of being well defined and less likely to elicit adverse reactions, including autoimmune responses. Here, we identify several antigens that, when used as part of a GP-based vaccine formulation, protect mice against lethal cryptococcal challenge. Cda2 (also known as MP98) was originally identified as the antigen that stimulated a T cell hybridoma generated from immunized mice (16). Subsequent studies using a peptide–MHC-II tetramer containing a 13-amino-acid peptide from Cda2 revealed that 6.5% of Th cells stained with the tetramer 14 days postinfection with *C. neoformans* strain KN99 (32). In addition to being identified on our biased screen of immunostimulatory proteins, Cda2 was the fifth most common protein found in the protective alkaline extracts (15). The two other definitive Cdas, Cda1 and Cda3, were also tested in our vaccine model. Each of the three recombinant CDAs, when delivered in a GP-based vaccine, protected at least one mouse strain against *C. neoformans* challenge, with rCda1 and rCda2 being the most promising of the antigens. Interestingly, it was recently shown that vaccination with a strain of *C. neoformans* genetically deficient in CDAs protected mice against a subsequent lethal *C. neoformans* challenge (9). Taken together, these data highlight that while the CDAs are promising vaccine candidates, other protective antigens exist.

One such protective antigen, identified as one of the top two hits in our unbiased screen (15), is Sod1. These results validate the unbiased approach, and we are presently testing the other abundant proteins identified in the alkaline extracts. While the CDAs have little to no homology with human proteins, Sod1 may not be an ideal candidate vaccine antigen due to its significant homology with human superoxide dismutase (SOD). However, the immunoinformatic analysis supports the prospect that epitope mapping will identify a protective region within the antigen that has minimal homology with human antigens. Indeed, future studies will seek to find such regions within the various protective antigens and then test the efficacy of chimeric antigens containing the protective epitopes.

The recombinant proteins were made in *E. coli* and thus are likely lacking in glycosylation. Cryptococcal N-linked and O-linked glycans generally are terminally mannosylated. Upon infection, the mannose groups are recognized by C-type lectin receptors with affinity for mannose (22). Notably, this interaction of mannosylated proteins with host mannose receptors augments immunity. Diminution of T cell responses was observed following chemical deglycosylation of cryptococcal mannoproteins or by blocking mannose receptors with mannan (33, 34). In addition, Cda2-specific cytokine production was greater when cells were stimulated with recombinant Cda2 (rCda2) produced in the yeast *Pichia pastoris*, compared with *E. coli*-derived protein (35). Nevertheless, in the present study, the mice were protected following vaccination with proteins made in *E. coli*. We posit this is because the antigens are encased in GPs that are phagocytosed by Dectin-1 and complement receptors (24), obviating the participation of mannose receptors. Nevertheless, it would be interesting to compare responses of native and unglycosylated cryptococcal antigens encased in GPs.

A challenge in the development of vaccines designed to stimulate T cell-mediated protection is the diversity of MHC alleles within the human population (19). We tested our GP-based vaccines using three different mouse strains: C57BL/6, BALB/c, and DR4. We found that for a given antigen, the degree of observed protection varied as a function of mouse strain. The importance of MHC-II is evident comparing C57BL/6 and DR4 mice; GP-Cda2 and GP-Sod1 were partially protective vaccines in C57BL/6, but not DR4 mice. These mice share genetic backgrounds, with the exception that in transgenic DR4 mice, native MHC class II molecules have been replaced by human HLA-DR4 (36). GP-Cda1 and GP-Cda2 vaccines afforded complete or nearly complete protection in BALB/c mice, with many of the mice having undetectable CFU in the lungs at the time of euthanasia. Moreover, the GP-Cda3 vaccine, which was not protective in C57BL/6 and DR4 mice, was partially protective in BALB/c mice. As shown in Fig. S3, the predicted MHC-II binding affinity of individual sequential peptides within each protein varies considerably between the MHC-II alleles of BALB/c and C57BL/6 and DR4. Similar differences may be expected between individual human MHC-II alleles; the permuted average of the predicted binding of each peptide across multiple human MHC-II alleles provides an indication (Fig. S3) of which mouse epitopes may be most likely to elicit a comparable result in the human population. However, taken together, these results suggest that a successful cryptococcal vaccine that stimulates T cell protective responses in humans will require a combination of antigens or epitopes.

Pathology and CFU data demonstrate that vaccinated mice are able to mount an inflammatory response during the first week of infection and that this inflammatory response is correlated with a reduction in the fungal burden compared with challenged mice that received control vaccine. At day 7, vaccinated mice had 3- to 8-fold fewer CFU in the lung than control mice. By 14 days postinfection, the inflammatory responses in control and vaccinated mice are similar, yet CFU continue to rise in control mice while plateauing or falling in the vaccinated animals. This suggests there are differences in the qualitative nature of the inflammatory response not detected on H&E staining. We have shown that GP-based vaccines promote robust antigen-specific antibody, Th1, and Th17 responses (15, 20, 21). Importantly, Th1 and Th17 recall responses to the lungs occur following pulmonary challenge of vaccinated mice (15). Ongoing work is directed at further defining the mechanisms responsible for the protection observed with the GP-based recombinant protein vaccines.

It is estimated that *C. neoformans* and *C. gattii* diverged between 16 and 160 million years ago (37). Both species share major virulence determinants, although a comparison of their genomes reveals only 86% identity (37–39). Differences in immunopathology have been noted in experimental murine models when comparing infections with the two species (38, 40, 41). Thus, it was of interest to see whether vaccines that protected against *C. neoformans* would provide cross-protection in *C. gattii* models of infection. Remarkably, we found vaccination with GP-Cda1 and GP-Cda2 provided almost identical levels of protection against both cryptococcal species in C57BL/6 mice. However, in BALB/c mice, the same vaccines provided robust protection against challenge with *C. neoformans* but no protection against *C. gattii*. Comparative epitope mapping of *C. neoformans* and *C. gattii* is under way to determine if differences in epitopes engendered by the 35- to 42-amino-acid differences in each protein assist in identifying the basis for protection. The cryptococcal strains we used, KN99 and R265, are hypervirulent and thus provide a stringent test for any vaccine; future studies also will examine epitope differences in additional cryptococcal strains that commonly cause disease in humans.

Using the model antigen ovalbumin encapsulated in GPs, we previously demonstrated robust CD4^+^ T cell and antibody responses occur even after immunization with submicrogram doses of antigen (21). Our new studies extend these findings to demonstrate the utility of using the GP-based delivery system as an effective tool for vaccine antigen discovery. Four of the six antigens tested, when administered as a GP-based vaccine, provided significant protection in at least one mouse model. We are currently using this approach to identify other protective antigens based on our biased and unbiased screens. Eventually, the antigens must be tested in human systems. Moreover, as the greatest risk factor for cryptococcosis is T cell dysfunction (7), we hypothesize that a successful cryptococcal vaccine for use in humans will need to elicit not only T cell responses to protein antigens but also antibody responses. Regarding the latter, antibody to capsular and cell wall carbohydrates has been shown to be protective in some models of cryptococcosis (13, 14, 42). Using the GP delivery system, we are currently testing whether the addition of GXM conjugated to a protein carrier will boost protection in mouse models.

## MATERIALS AND METHODS

### Chemicals and culture media.

Chemical reagents were from Thermo Fisher Scientific (Pittsburgh, PA), unless stated otherwise. The media used for culturing of *Cryptococcus* were YPD (Difco yeast extract, Bacto peptone, dextrose, with and without 2% agar) and Sabouraud dextrose agar Emmons (Remel). *E. coli* strains TOP10 and BL21 were cultured at 37°C in LB broth or agar. Transformed strains of BL21 were used for protein expression by being cultured for 18 h at 30°C with shaking in Overnight Express Instant TB medium (EMD Millipore, Billerica, MA). Antibiotic selection was with ampicillin (100 μg/ml) or kanamycin (100 μg/ml).

### Strains of *Cryptococcus*.

*C. neoformans* var. *grubii* strains KN99 (43) and cap59 (44) and *C. gattii* strain R265 (45) were maintained as glycerol stocks at −80°C and initially cultured on YPD agar. To prepare *Cryptococcus* for *in vivo* challenge studies, KN99 and R265 were cultured in liquid YPD for 15 to 22 h at 30°C with shaking. Yeast cells were then harvested by centrifugation, washed once with phosphate-buffered saline (PBS), counted using a hemocytometer, and finally suspended in PBS at 2 × 10^5^ cells/ml. CFU were determined on Sabouraud dextrose agar.

### Expression of cryptococcal proteins in *E. coli*.

Six *C. neoformans* strain H99 proteins were expressed in *E. coli* (Table 1) for subsequent testing as vaccine candidates. When present, cDNA was deleted of regions coding for a signal peptide and C terminus rich in serine and threonine and the GPI anchoring motif. Five proteins were expressed following cloning of PCR-amplified cDNA in pET200/D-TOPO (Invitrogen), and for the sixth protein its cDNA was synthesized and cloned in pET19b by GenScript (Piscataway, NJ). Both vectors are designed to fuse vector-encoded sequence that contains a His tag to the N terminus of the cloned cDNA.

Briefly, strain cap59 was cultured in YPD at 30°C with shaking to the mid-log phase. Approximately 10^8^ cells were collected by centrifugation in a 2.0-ml microcentrifuge tube, suspended in 0.7 ml RLT Plus buffer containing β-mercaptoethanol, and homogenized using 0.6 ml of 0.7-mm zirconium beads (BioSpec Products, Bartlesville, OK). Purification of RNA from the homogenate was performed according to the manufacturer’s instructions (RNeasy Plus minikit; Qiagen, Hilden, Germany). Purified RNA was used to make cDNA using the iScript cDNA synthesis kit (Bio-Rad Laboratories, Hercules, CA). The cDNA was then used as the template for amplification by PCR with Platinum Pfx DNA polymerase (Invitrogen) with gene-specific primers designed for directional cloning. Each PCR product was purified using a QIAquick PCR purification kit (Qiagen) and then ligated to pET200/D-TOPO according to the manufacturer’s instructions that accompanied the Champion pET 200 Directional TOPO expression kit (Invitrogen). The ligated DNA was transformed into *E. coli* TOP10 cells and plated for single-colony isolation. Several colonies were screened by PCR using PCR SuperMix (Invitrogen) and gene-specific primers. PCR products of the predicted size following electrophoresis in agarose were confirmed, and plasmid DNA was purified from select clones grown in 4 ml LB-kanamycin medium using a QIAprep spin miniprep kit (Qiagen). DNA then was sequenced (GeneWiz, South Plainfield, NJ) using vector forward and reverse primers. Plasmids confirmed to have the expected cDNA sequence ligated correctly were transformed into *E. coli* BL21 cells for protein expression. Glycerol stocks of *E. coli* were made for storage at −80°C.

### Purification of proteins.

Each recombinant BL21 strain was grown in 50 ml of Overnight Express medium with antibiotic in 250-ml baffled culture flasks (Bellco Glass, Vineland, NJ) at 30°C for 18 h. Cultures were inoculated with approximately 25 μl of −80°C glycerol stock. The buffers used in purification of protein on His·Bind resin were recommended by the manufacturer (EMD Millipore). Cells were harvested by centrifugation at 6,000 × *g* for 10 min, suspended in 5 ml binding buffer (BB: 0.5 M NaCl, 20 mM Tris-HCl, 5 mM imidazole [pH 7.9]), sonicated for 30 s, and collected by centrifugation. The cell pellets were suspended in 5 ml BB containing 6 M urea and sonicated again. Following centrifugation, the supernatant was collected and centrifuged at 20,000 × *g* for 5 min to remove residual insoluble material. Clarified supernatants from two 50-ml cultures were combined (ca. 9 ml) and applied to a 3-ml bed volume of His·bind resin that had been equilibrated with BB plus 6 M urea. The column was washed with 15 ml BB–6 M urea and 10 ml wash buffer (0.5 M NaCl, 20 mM Tris-HCl, 60 mM imidazole, 6 M urea [pH 7.9]), and protein was eluted with a mixture of 0.5 M NaCl, 20 mM Tris-HCl, 1 M imidazole, and 6 M urea (pH 7.9). The eluate was collected in 1-ml fractions, which were each analyzed by SDS-PAGE to determine which fractions to pool—typically 3 to 4 fractions. The purified protein was dialyzed against 6 M urea–20 mM Tris-HCl and concentrated to 10 mg/ml using Amicon Ultra-15 centrifugal filters (10-kDA cutoff; Merck Millipore, Cork, Ireland). All procedures following the growth of cells were at 22°C. Protein determinations were by the bicinchoninic acid assay. Samples of concentrated protein were stored at −80°C. For SDS-PAGE, proteins were separated on 4 to 20% gradient gels (BioRad) in Tris-glycine-SDS running buffer and stained with Coomassie (Expedeon, Ltd., Cambridgeshire, United Kingdom); protein standards were from BioRad.

### GP vaccines.

*Saccharomyces cerevisiae* cells were purified into GPs following a series of hot alkali, organic, and aqueous extraction steps, as described previously (20, 21, 46, 47). The final GP vaccine consisted of recombinant protein, mouse serum albumin (MSA; Equitech-Bio, Kerrville, TX) and yeast RNA (yRNA; Sigma-Aldrich, St. Louis, MO) complexed within the glucan shells. Each recombinant protein at 10 mg/ml in 6 M urea–20 mM Tris-HCl was loaded into GPs and complexed with MSA and yRNA as described previously (20, 21, 46). Vaccines were diluted in sterile 0.9% saline for injection to deliver 10 μg of antigen and 25 μg of MSA complexed with yRNA in 200 μg GPs (approximately 10^8^ particles) per 0.1-ml dose. Control vaccine was prepared identically with GPs loaded with MSA and yRNA, but without recombinant protein. Vaccines were stored in 0.6-ml aliquots at −80°C and briefly vortexed prior to use. Vaccine formulations were quality controlled by manual counting of GPs per milliliter using a hemocytometer, microscopically assessing for intact GPs with a phase-distinct protein-yRNA complex. Vaccines were also extracted and analyzed by 10% SDS-PAGE to verify loading of antigens and MSA (15).

### Mice.

Six-week-old female C57BL/6 and BALB/c mice were obtained from either Charles River Laboratories, Inc. (Kingston, NY), or The Jackson Laboratory (Bar Harbor, ME). The Abb knockout/transgenic HLA-DR4 (DR4) mice were from Taconic Biosciences (Hudson, NY). Mice were bred and housed in a specific-pathogen-free environment in the animal facilities at the University of Massachusetts Medical School. Vaccinations commenced when the mice were 6 to 10 weeks of age; both male and female mice were used. All animal procedures were carried out under a protocol approved by the University of Massachusetts Medical School Institutional Use and Care of Animals Committee.

### Vaccination and challenge studies.

The GP-based vaccines (0.1-ml dose) were administered three times at 2-week intervals as a subcutaneous injection at the midline of the abdomen, as described previously (20, 21). Two weeks following the third vaccination, the mice were challenged with 10^4^ CFU of *C. neoformans* strain KN99 or *C. gattii* strain R265 in 50 µl of PBS. Mice were anesthetized with 2% isoflurane (Piramal Health Care, Andrah Pradesh, India) in a VetEquip laboratory animal anesthesia system (VetEquip, Livermore, CA) and inoculated orotracheally. For survival studies, mice were monitored twice daily and euthanized if they developed advanced signs of disease, including ataxia, listlessness, and failure to groom. At the termination of the study, the surviving mice were euthanized, and their lungs and brains were removed. Lungs were weighed, and the organs were homogenized in PBS containing 100 U/ml penicillin and 100 µg/ml streptomycin (lungs in 4 ml and brains in 2 ml) using an Omni TH tissue homogenizer and plastic homogenizing probes (Omni, Kennesaw, GA). Undiluted and diluted homogenates were plated on Sabouraud medium and incubated at 30°C for 2 to 3 days, at which time fungal CFU were enumerated. In other studies, mice were euthanized at various times after fungal challenge to enumerate CFU. The lower limit of detection was 20 CFU. Lungs with no CFU were arbitrarily assigned a value of 20 CFU.

### Histology.

Lungs from euthanized mice were inflated with 0.5 ml 4% paraformaldehyde in PBS and then excised. Following embedding in paraffin, 5-μm sections obtained from each of five lobes were mounted on glass slides and stained with H&E or mucicarmine (Sigma-Aldrich). A pathologist, blind as to experimental group, then scored the slides on a scale of 0 to 3 for pulmonary inflammation and fungal burden as shown in Fig. S2.

### Immunoinformatics.

Each of the six proteins was analyzed as previously described to evaluate the distribution of B cell linear epitopes, MHC binding for human and murine alleles (48, 49), and the presence or absence of possible suppressive T cell epitope motifs (50, 51). In addition, both wild-type and recombinant sequences of each candidate protein were evaluated to determine the content of high-frequency T-cell-exposed motifs relative to all other proteins in the *C. neoformans* proteome (51). More details are presented following the legend to Fig. S2.

### Statistics.

Data were analyzed using GraphPad Prism, version 6.0 (GraphPad Software, Inc., La Jolla, CA). Kaplan-Meier survival curves were compared using the Mantel-Cox log rank test. The unpaired two-tailed *t* test was used for comparisons of two groups with the Bonferroni correction applied for multiple comparisons. The one-way analysis of variance (ANOVA) with the Tukey multiple-correction test was used to compare more than two groups.
