# Supplementary material for: Vaccination with Recombinant Cryptococcus Proteins in Glucan Particles Protects Mice against Cryptococcosis in a Manner Dependent upon Mouse Strain and Cryptococcal Species
Source: mBio. 2017 Nov 28;8(6):e01872-17. doi: 10.1128/mBio.01872-17 (PMC5705919; doi:10.1128/mBio.01872-17)
Supplement: FIG S1 [file mbo006173613sf1.pdf]

Supplementary Figure S1.

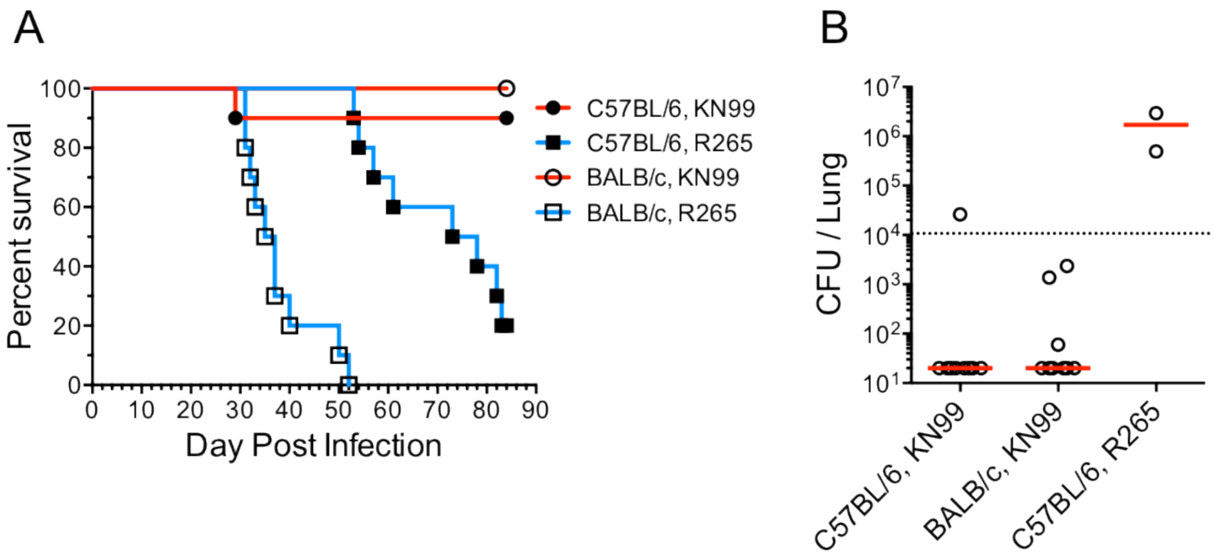

FIG S1 Survival of C57BL/6 and BALB/c mice vaccinated with Cda1 and Cda2 and challenged with *C. neoformans* strain KN99 or *C. gattii* strain R265. Mice were vaccinated with a mixture of Cda1 and Cda2 as in Fig. 2C, except the vaccination dose for each antigen was 6 μg. The challenge dose of KN99 and R265 was 10^4 CFU. (A) Survival curves of vaccinated and challenged mice. Data are from two independent experiments with 4 or 5 mice per group. P<0.001 comparing C57BL/6 KN99 with C57BL/6 R265. P<0.001 comparing BALB/c KN99 with BALB/c R265. Unvaccinated controls all died between 16 and 33 days post-infection (data not shown). (B) Lung fungal burdens of mice that survived for 12 weeks. For panel B, the dotted line identifies CFU of KN99 and R265 challenge; the median CFU per lung for each group is indicated by a solid line.
