# Supplementary material for: Vaccination with Recombinant Cryptococcus Proteins in Glucan Particles Protects Mice against Cryptococcosis in a Manner Dependent upon Mouse Strain and Cryptococcal Species
Source: mBio. 2017 Nov 28;8(6):e01872-17. doi: 10.1128/mBio.01872-17 (PMC5705919; doi:10.1128/mBio.01872-17)
Supplement: FIG S2 [file mbo006173613sf2.pdf]

Supplementary Figure S2.

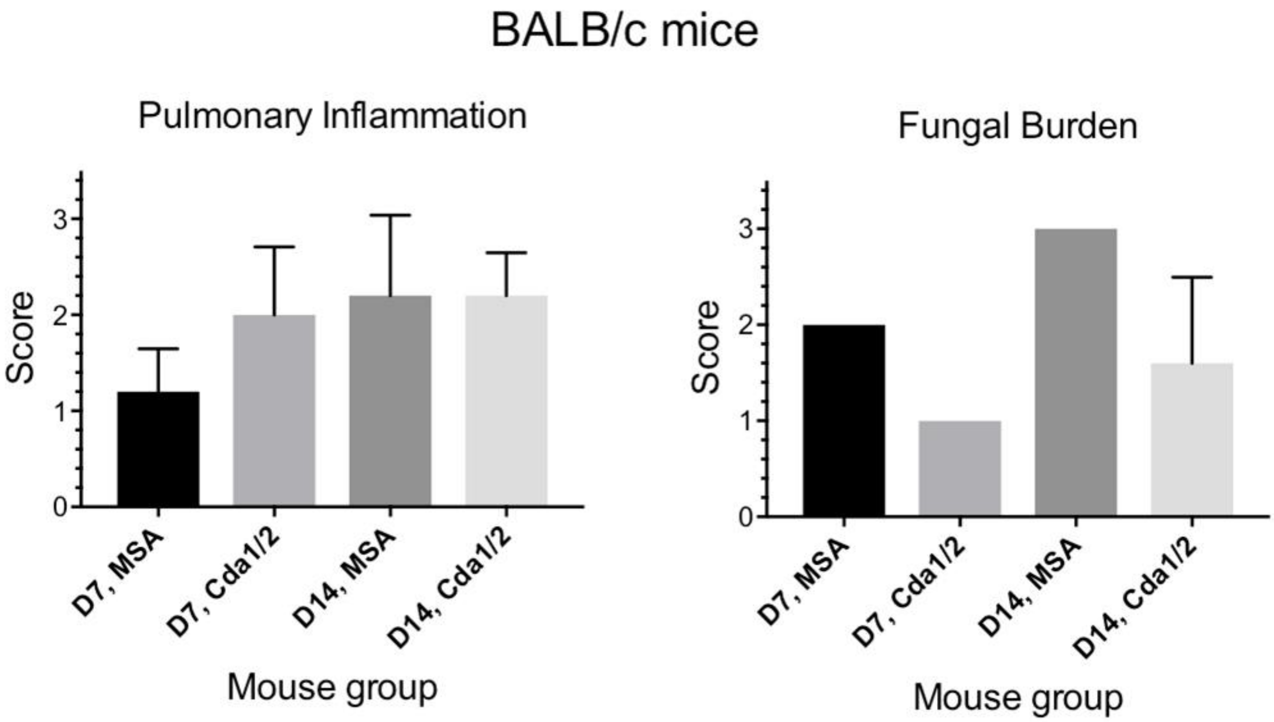

Pulmonary Inflammation	Score
No inflammation present	0
< 33% of lung inflamed	1
33% - 66% of lung inflamed	2
66% -100% of lung inflamed	3
**Fungal Burden**	**Score**
No organisms seen	0
<33% of alveoli or airways contain organisms	1
33% - 66% of alveoli or airways contain organisms	2
66 - 100% of the alveoli or airways contain organisms	3

Pulmonary inflammation and fungal burden seen on pathology. BALB/c mice were vaccinated three times with a 10 μg/dose each of GP-Cda1 and GP-Cda2 (Cda1/2) and then challenged with 10^4 CFU of *C. neoformans* strain KN99. Control mice received GP-MSA (MSA). At 7 and 14 days post-challenge, mice were euthanized, and stained slides prepared from sectioned lungs were scored by a pathologist blinded as to group. n= 5 mice/group. For fungal burdens, $p<0.05$ and $p<0.001$ comparing MSA with Cda1/2 at D7 and D14, respectively.
