# Supplementary material for: Vaccination with Recombinant Cryptococcus Proteins in Glucan Particles Protects Mice against Cryptococcosis in a Manner Dependent upon Mouse Strain and Cryptococcal Species
Source: mBio. 2017 Nov 28;8(6):e01872-17. doi: 10.1128/mBio.01872-17 (PMC5705919; doi:10.1128/mBio.01872-17)
Supplement: FIG S3 [file mbo006173613sf3.pdf]

Supplementary Figure S3. Comparison of B cell linear epitopes and human and murine MHC II binding patterns.

FIG S3A

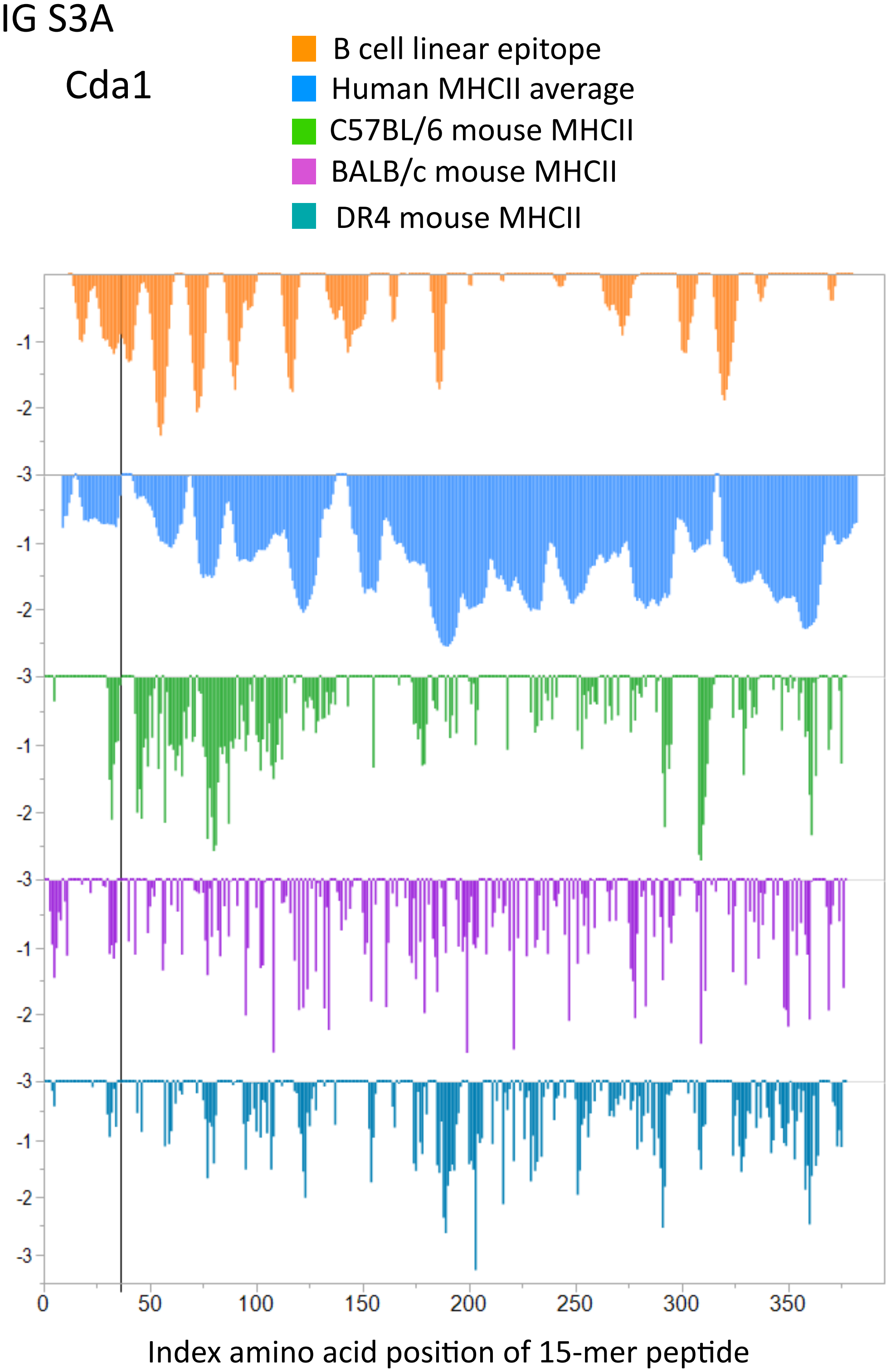

FIG S3B

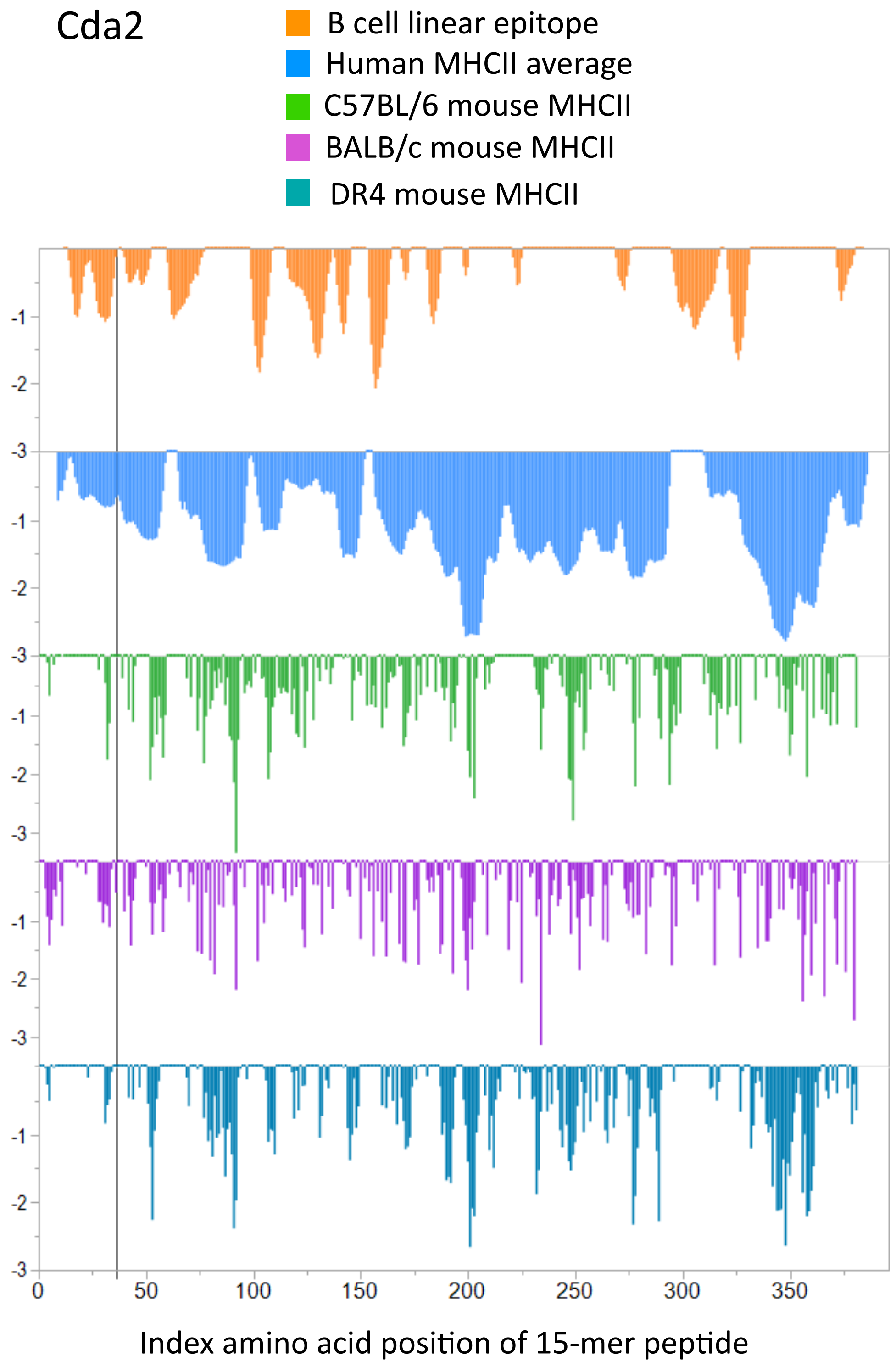
Cda2
B cell linear epitope
Human MHCII average
C57BL/6 mouse MHCII
BALB/c mouse MHCII
DR4 mouse MHCII
-1
-2
-3
0
50
100
150
200
250
300
350
Index amino acid position of 15-mer peptide

FIG S3C

Cda3

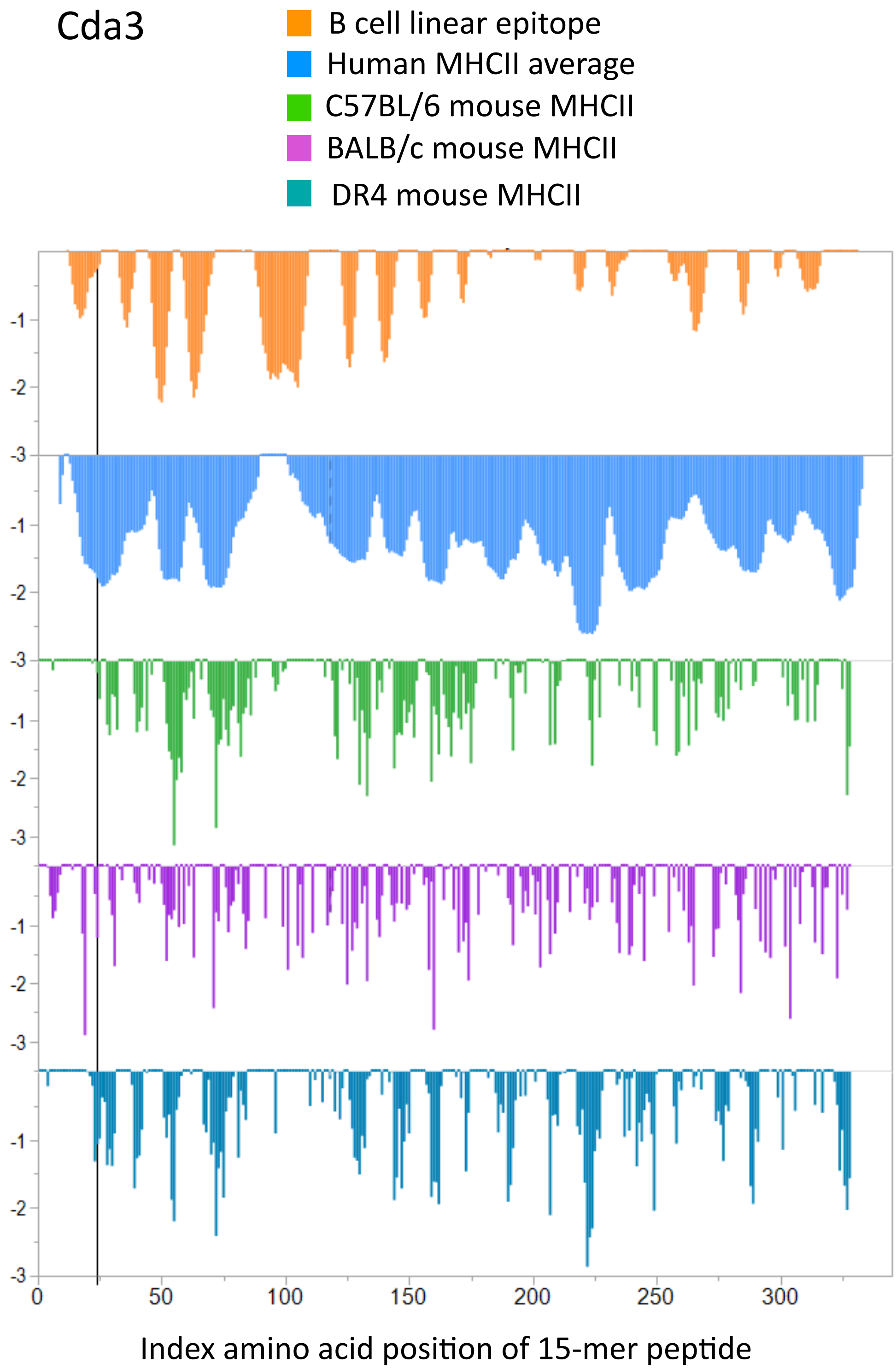

B cell linear epitope
Human MHCII average
C57BL/6 mouse MHCII
BALB/c mouse MHCII
DR4 mouse MHCII
-1
-2
-3
0
50
100
150
200
250
300
Index amino acid position of 15-mer peptide

FIG S3D

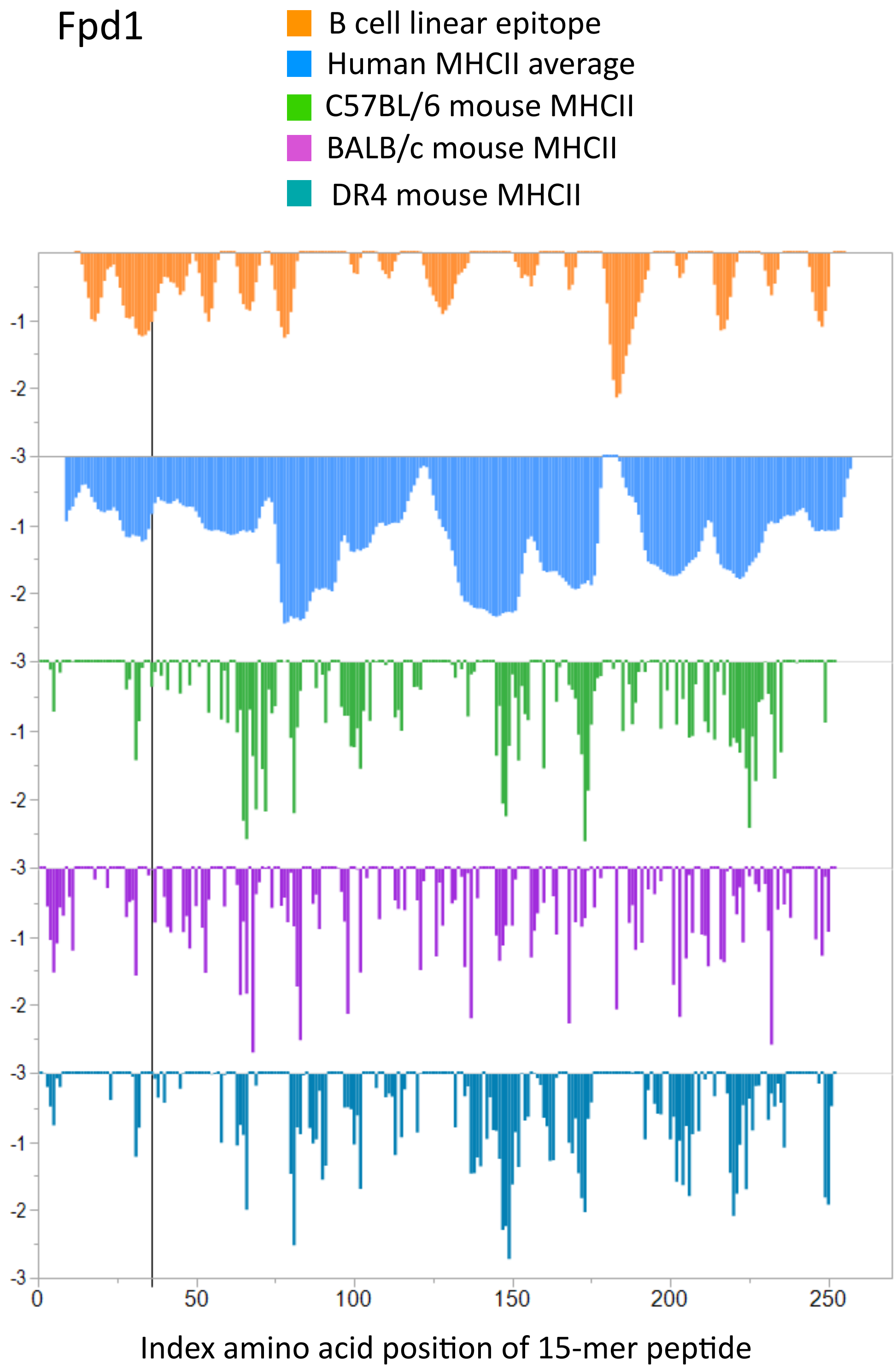

Fpd1
B cell linear epitope
Human MHCII average
C57BL/6 mouse MHCII
BALB/c mouse MHCII
DR4 mouse MHCII
-1
-2
-3
0
50
100
150
200
250
Index amino acid position of 15-mer peptide

FIG S3E

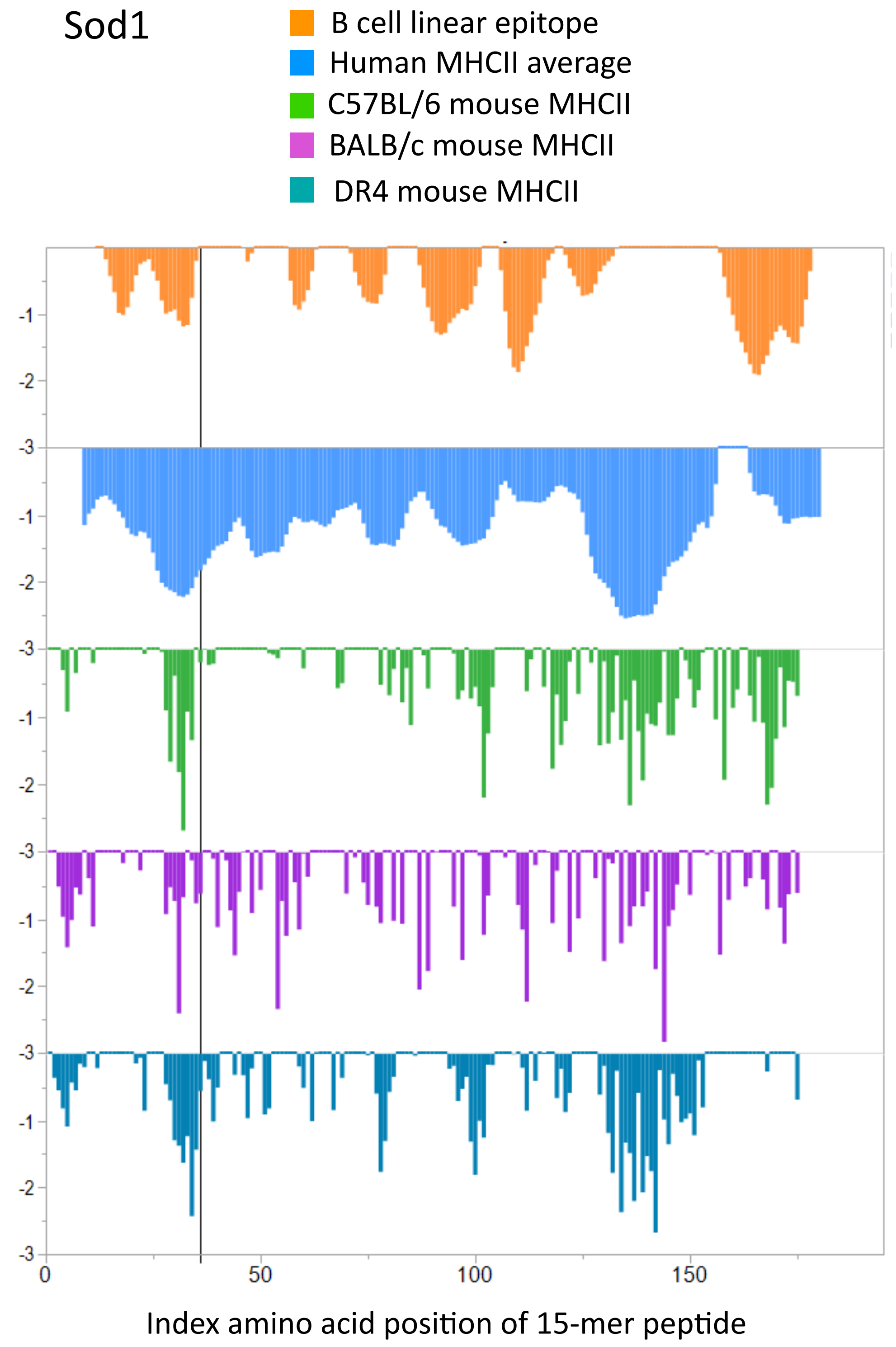

Sod1
B cell linear epitope
Human MHCII average
C57BL/6 mouse MHCII
BALB/c mouse MHCII
DR4 mouse MHCII
-1
-2
-3
0
50
100
150
Index amino acid position of 15-mer peptide

FIG S3F

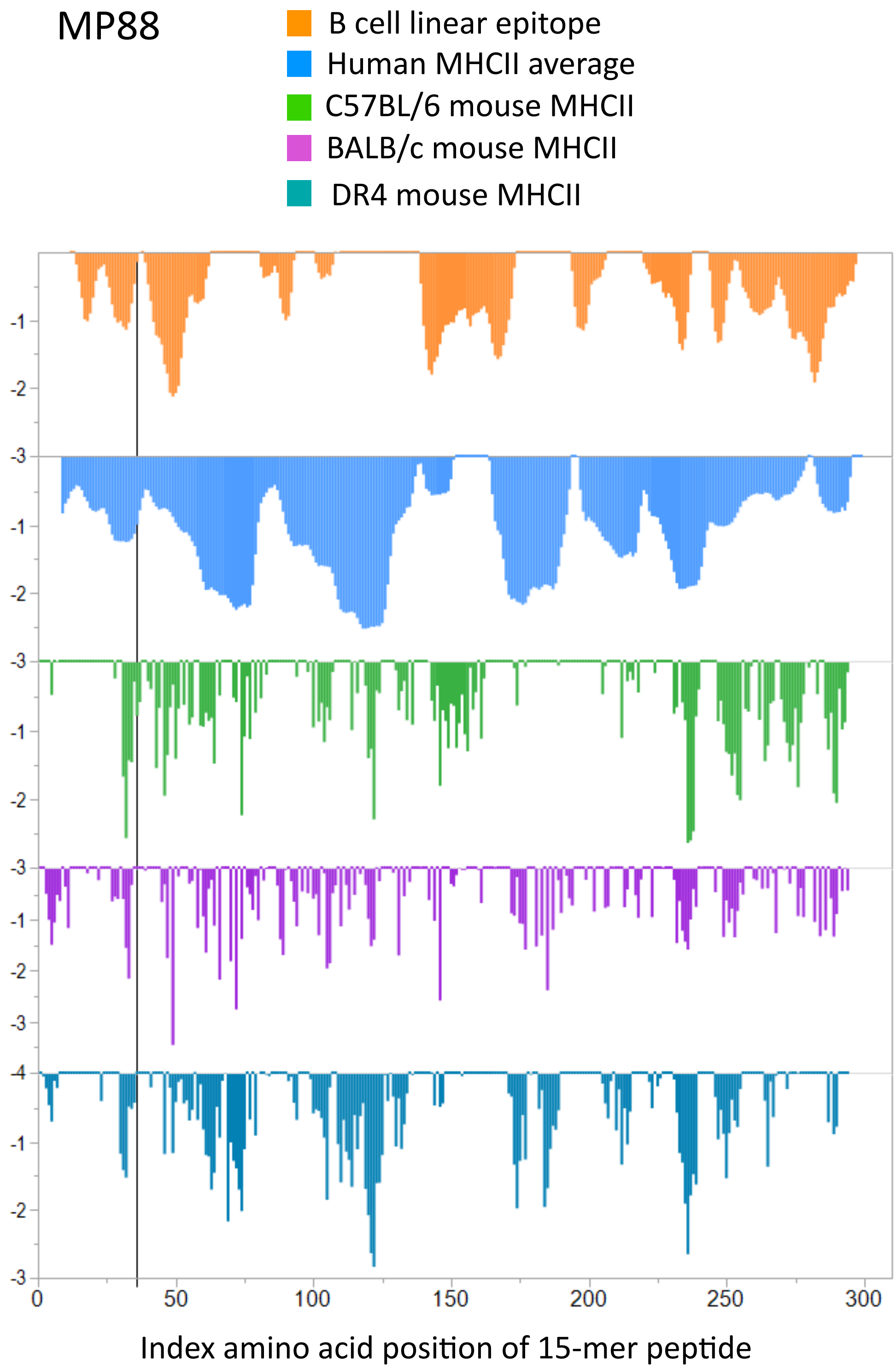
MP88
B cell linear epitope
Human MHCII average
C57BL/6 mouse MHCII
BALB/c mouse MHCII
DR4 mouse MHCII
-1
-2
-3
-1
-2
-3
-1
-2
-3
-1
-2
-3
-4
-1
-2
-3
0
50
100
150
200
250
300
Index amino acid position of 15-mer peptide

Figure S3. For each protein tested as a candidate vaccine antigen, the probability of participation in a B cell linear epitope and predicted MHC-II binding affinities of sequential 15-mer peptides are shown in the five panels. Panels should be read from top to bottom. Panel 1 shows the probability of linear B cell epitopes for both mice and humans. Panel 2 shows the permuted human MHC-II binding, smoothed across 16 DRB alleles. Panel 3 shows MHC-II binding for allele H-2-IAb (C57BL/6 mice). Panel 4 shows MHC-II binding for allele H-2-IAd (BALB/c mice). Panel 5 shows MHC-II binding for human allele DRB1-0401 found in transgenic mouse strain DR4. y-axis units are the inverted standard deviation of linear B cell epitope probability, and for MHC binding panels, they are standard deviation units below the mean of the natural log binding affinity (ln of the 50% inhibitory concentration [$lnIC_{50}$]) for that protein. The vertical black line in each set of panels indicates the boundary between vector sequence that includes the His tag and each Cryptococcus protein sequence. The epitope predictions illustrated in panels A to F were generated using the ioGenetics' EigenBio platform. The EigenBio algorithms are written on JMP (SAS Institute). The rationale underlying the predictions is further described in several publications by R. D. Bremel and E. J. Homan as cited in Text S1 in the supplemental material.

Text S1: Methods specific to Figure S3. To determine MHC binding, predictions of the natural logarithm of binding affinity (ln IC50) are produced by an ensemble of neural network (NN) equations. Equation sets are available for 28 class II human alleles (16 DRB, 6 DP, and 6 DQ) and 3 class II mouse alleles (IAb, IAd, Ag7). The sizes of the training sets of experimental peptide binding reactions used to train the NN ranges from a minimum of 100 to over 4000 peptides per allele. Primary amino acid sequences of the peptides used for training as well as the sequences of the proteins analyzed are transformed into numerical vectors consisting of the first three principal components of amino acid physical properties (PCAA). The PCAA were derived from a number of different independent studies in the literature where a variety of different physical properties were characterized. The principal components are most obviously associated with polarity, size, and electronic character of amino acids (but are each abstract variables not identical to those parameters). Training of the NN to produce the equation ensembles was done by a bootstrap aggregation approach (known as "bagging") and a 5K-fold cross validation procedure. The use of ensembles developed by the bagging approach means that both a mean and standard deviation of the ln IC50 estimates for each peptide can be computed. The MHC binding affinity predictions for any peptide are then based on the average probabilities of the ensembles. The standard deviations are different for each peptide and are

related to the strength of training for a particular combinatorial group of amino acids and range from 0.5 – 1.2 natural log units, a level of variation quite comparable to experimental measurements of binding affinities. To remove scale effects, and to make valid statistical comparisons within and between alleles, the ln IC50 values are standardized within each protein molecule to zero mean and unit variance. In addition to removing any scale effects due to differences among alleles, this process also has the effect of ranking the peptides within a protein by their respective binding affinities. The standardization is done using a Johnson Sb distribution algorithm.

Predictions of B-cell epitope (BEPI) contact points are produced by a binary classifier neural network using the PCAA of the primary amino acid sequence of the protein, as is described above. The classifier was trained with the same epitope training set used by web accessible classifier BEPI-pred (5) and shows a Pearson correlation coefficient with BEPI-pred of 0.93. The BEPI classifier produces a probability of an amino acid in the center of a 9-mer peptide of being bound by a B-cell receptor – effectively a B-cell contact point. It does not predict epitopes per se, but rather individual linear components of what might be a more complex 3-dimensional epitope. We have shown that the AROC (concordance) of prediction against X-ray crystallographically defined B-cell contact points to be 0.87. In addition, we have found the predictions to be highly correlated with / concordant to both crystallographic B-factors and amino acid side chain solvent accessibility.

In the Supplemental Figures 3A-F, the top panel in each figure shows the predicted probability of each 9-mer peptide comprising a linear B cell epitope contact point. Each orange bar making up the display is scaled on a Y axis that shows the probability of binding as the inverse of the standard deviation below the mean for all peptides in the protein. The inverse is used to allow plotting alongside the MHC binding predictions. Thus, the longest orange bars indicate the highest probability that the peptide is part of a B cell linear epitope.

The second panel shows the permuted average binding affinity predicted for all combinations of human DRB MHC II alleles at each sequential 15-mer peptide (with a single amino acid displacement) across the protein. The Y axis is in units of standard deviation below the mean predicted binding for all peptides in the protein. Thus, this provides an overview of the peptides in which highest or lowest MHC II binding occurs in the human population at large; where the lower figures indicate highest binding. Because it is the average of 16 DRB alleles it shows as a smoother curve than the individual peptides for the single mouse alleles below.

In the lower three panels in each graphic, separate outputs were generated to identify the predicted binding of sequential 15-mer peptides across each protein for the murine MHC II alleles H2-I-Ab (C57BL/6 mouse) and H2-I-Ad (BALB/c mouse), as well as for the human allele DRB1:0401). Predicted binding affinity for each peptide is indicated by an individual bar where the Y axis is in standard deviation units below the mean as above.

Supplemental Methods References.

1. Bremel RD, Homan EJ. 2010. An integrated approach to epitope analysis II: A system for proteomic-scale prediction of immunological characteristics. Immunome Res 6:8.

2. Bremel RD, Homan EJ. 2010. An integrated approach to epitope analysis I: Dimensional reduction, visualization and prediction of MHC binding using amino acid principal components and regression approaches. Immunome Res 6:7.

3. Bremel RD, Homan EJ. 2013. Recognition of higher order patterns in proteins: immunologic kernels. PLoS One 8:e70115.

4. Bremel RD, Homan EJ. 2014. Frequency Patterns of T-Cell Exposed Amino Acid Motifs in Immunoglobulin Heavy Chain Peptides Presented by MHCs. Front Immunol 5:541.

5. Larsen JE, Lund O, Nielsen M. 2006. Improved method for predicting linear B-cell epitopes. Immunome Res 2:2.
